# Population-based cross-sectional analysis of cannabis use among Kentucky adults, 2020–21

**DOI:** 10.1186/s42238-024-00251-x

**Published:** 2024-12-20

**Authors:** Sydney Shafer, Gunnar Kennedy, W. Jay Christian

**Affiliations:** 1 University of Kentucky, Lexington, US; 2 Kentucky Department for Public Health, Lexington, US

**Keywords:** Cannabis, Marijuana, Kentucky, Surveillance, Epidemiology

## Abstract

**Purpose:**

We conducted this study to assess cannabis use rates in the state of Kentucky relative to socioeconomic, demographic, and geographic factors, as well as reasons for use and modes of use, before the legal medical marijuana market commences in 2025.

**Methods:**

We pooled Kentucky Behavioral Risk Factor Surveillance System (BRFSS) data for 2020–2021 and used weighted responses for all analyses. We estimated current cannabis use (at least once in the past 30 days), and heavy use (at least 20 of the past 30 days) prevalence rates for Appalachian, Delta, and Central geographic regions of Kentucky. We tabulated descriptive statistics and used multivariable logistic regression to identify characteristics of individuals who used cannabis.

**Results:**

The prevalence of cannabis use was lower in Kentucky (10%) than nationally (about 13%). Of those who used cannabis, 42% used it daily or near daily. Those who were male, ages 18–34, never married, black, less than HS education, lower household income, and lived in the Central region were more likely to use cannabis. Among those who used cannabis, mode of use varied somewhat among age groups, education levels, income groups, and marital status, but smoking was most common—78% overall. About 33% reported using cannabis for recreation alone, 24% for medical reasons alone, and 43% for both reasons.

**Conclusion:**

Despite the illegal status of cannabis in Kentucky, its use is common across population sub-groups. A large proportion of Kentuckians using cannabis do so daily or near daily, and most for a medical purpose. Smoking, however, remains the most common mode of use.

## Introduction

Cannabis is the most frequently used federally illicit substance in the United States (US), with rates continuing to rise as the state-level legalization of both medical and recreational use becomes more widespread (Groce 2018). Governor Andy Beshear signed legislation to legalize medical cannabis use in Kentucky beginning January 1st, 2025 (Kentucky Medical Cannabis Program 2023). Until the medical marijuana program begins in Kentucky, a 2022 Executive Action by Gov. Beshear provides a conditional pardon to protect people who use cannabis for medical purposes from prosecution (Kentucky Medical Cannabis Program 2023). To be eligible for the pardon, patients must have a written certification from a doctor stating they have been diagnosed with one of 21 conditions (Kentucky Medical Cannabis Program 2023). Some of the conditions listed eligible for the pardon in Kentucky are AIDS, cancer, epilepsy, multiple sclerosis, and muscular dystrophy. This pardon does not guarantee that those who use cannabis for medical reasons won’t be arrested for marijuana possession.

Cannabis use presents a range of risks and benefits that are not yet fully understood. There has been increasing evidence detailing the efficacy of cannabis for some therapeutic purposes, including chronic and neuropathic pain management, relief from chemotherapy-induced nausea and vomiting, and for epilepsy management (Keyhani et al 2018). These medical uses are among the driving forces behind the push for legalization and broader social acceptance, with 90% of Kentucky adults supporting legalization of medical cannabis (Exec. Order No. 2022–798, 2022). Risks include higher likelihood of cannabis use disorder and other adverse health conditions, particularly with high potency products and frequent consumption (Connor et al. 2021). Those with chronic conditions may use cannabis to minimize symptoms of their condition/s, otherwise known as self-medicating (Asselin et al. 2022).

The psychoactive component of cannabis is the cannabinoid Δ−9 tetrahydrocannabinol, or THC.

There are a wide range of products available containing varying levels of THC, such as flower (for smoking), food (edibles), drinks, concentrated waxes (for dabbing), and vaporizers (Schauer et al. 2020). Legal markets increase availability of different types of products, leading to more variation in the modes by which people consume cannabis. Different modes of consumption are associated with differing drug effects and outcomes. For example, edibles have a longer onset time than other modes of consumption and may produce more intense effects and a longer lasting high (Barrus et al. 2016). Although such products are not available for retail sale in Kentucky, it is likely that some residents will purchase them in other states and bring them home for later use.

Many of the negative health effects known to be associated with cannabis use are due to the mode of use, in particular smoking and vaping. Smoking cannabis irritates the airways and is associated with chronic bronchitis and other respiratory diseases (American Lung Association, 2023). There is some evidence of an additive effect of smoking tobacco and cannabis on symptoms of chronic bronchitis (Tashkin 2015). Vaping nicotine products and cannabis products can also cause respiratory health problems, particularly when those products are unregulated (Traboulsi et al. 2020). These respiratory health effects are especially concerning for Kentucky, a state known to have high rates of tobacco smoking and respiratory disease (Kentucky Public Health 2021).

There are more than 100 cannabinoids produced by the *Cannabis sativa* plant species other than THC. Cannabidiol, or CBD, is another cannabinoid frequently featured in retail products. CBD can be derived from either marijuana or hemp, with hemp-derived CBD being de facto legal under the Agriculture Improvement Act of 2018, commonly known as the 2018 Farm Bill (US Forest Service 2022). By law, hemp-derived products must contain less than 0.3% THC (FDA 2024). It is common for CBD to be included in the definition of cannabis, though it is important to analyze CBD and THC separately when examining their effects. There are other hemp-derived cannabinoids that have recently become popular, namely Δ−8 and Δ−10 THC. This study excludes CBD and other hemp-derived products in its definition of cannabis.

This study examined the overall prevalence of cannabis use in Kentucky, characteristics of people who use cannabis, frequency of use, reasons for use, and preferred mode of use. Since Kentucky has a high cigarette smoking rate (Holford et al 2023) and smoking cannabis is the mode in which it is most frequently consumed (Schauer et al 2020) we also compared prevalence of use in Kentucky to other states without legal cannabis of any kind. Understanding how Kentuckians are using cannabis before the legal medical marijuana market commences in 2025 is vital for assessing the public health implications of this major policy shift in the future.

## Methods

### Data source and sample

The Behavioral Risk Factor Surveillance System (BRFSS) survey is administered by the Centers for Disease Control and Prevention (CDC) and is the largest individual health behavior surveillance system in the United States. It is a cross-sectional annual telephone survey conducted in all 50 states, the District of Columbia, and three US territories (CDC 2023). The BRFSS consists of both core and optional modules to cover many topics regarding health-related risk behaviors and events, chronic health conditions, and use of preventive services. The core modules are asked by every state each year, and the optional modules used are selected by states and differ each year. Questions about cannabis use are in an optional “Marijuana Use” module that was used in Kentucky in 2020 and 2021. BRFSS data are publicly available without the geographic variable for county, but for this study we obtained the data set with county of residence under agreement with the KY Department for Public Health.

### Measures

The BRFSS includes this preamble for the marijuana use module: *The following questions are about marijuana or cannabis. Do not include hemp-based or CBD-only products in your responses*. Participants were asked: *During the past 30 days, on how many days did you use marijuana or cannabis?* Possible answers ranged from 0 to 30 days. Respondents who reported at least one day of cannabis use were classified as people who currently use cannabis, with those who reported at least 20 days of use classified as heavy users. Those who did not report a number of cannabis use days, coded as either “don’t know/not sure” or “refused,” were excluded from analysis.

Participants who reported any cannabis use were then asked: *What was the reason you used marijuana?* Possible answers were 1) for medical reasons (like to treat or decrease symptoms of a health condition), 2) for non-medical reasons (like to have fun or fit in), or 3) for both medical and non-medical reasons. Participants who reported using cannabis were also asked: *During the past 30 days, which of the following ways did you use marijuana the most often? Did you usually…* Possible answers were 1) smoke it (for example, in a joint, bong, pipe, or blunt), 2) eat it (for example, in brownies, cakes, cookies, or candy), 3) drink it (for example in tea, cola, alcohol), 4) vaporize it (for example, in an e-cigarette-like vaporizer or other vaporizing device), 5) dab it (for example, using waxes or concentrates), or 6) use it some other way. For this study, we collapsed these modes into four categories. Eating and drinking were combined into group “Eat/drink,” dabbing and vaping were combined into group “Concentrate,” and both “Smoke” and “Other” remained on their own.

We used responses to other questions routinely included in the BRFSS survey to determine participant demographics. These characteristics included: six-level imputed age category, imputed race/ethnicity, computed income categories, computed level of education completed, health plan coverage, marital status, calculated sex variable, and county code. We collapsed the response categories for some questions for these analyses due to low response rates. The six-level imputed age variable was reduced to three categories: 18 to 34, 35 to 54, and 55 + . We used the imputed race/ethnicity variable and combined multiple categories due to low response rates. We left the imputed values of “White, Non-Hispanic” and “Black, Non-Hispanic” as categories of their own, and combined the values for “American Indiana/Alaskan Native Non-Hispanic,” “Hispanic,” and “Other race, Non-Hispanic” into one category, “Hispanic/Other.” We also combined values for computed levels of highest educational attainment. “Did not graduate High School” and “Graduated High School” were combined into one category, “High School Graduate or Below.” The two categories left on their own are “Attended College or Technical School” and “Graduated from College or Technical School.” Six computed income response categories were collapsed into three: “Less than $25,000,” “$25,000–49,999,” and “$50,000 + .” Finally, marital status responses of “Married” and “Member of unmarried couple” were combined into “Married/Cohabitating.” “Separated”, “Divorced”, and “Widowed” were all combined into one value, and “Never Married” was left as a category of its own.

The BRFSS includes several questions related to both cigarette smoking and alcohol use, which were included as covariates. We used two questions to ascertain smoking status: 1) *Have you smoked at least 100 cigarettes in your entire life?* and 2) *Do you now smoke cigarettes every day, some days, or not at all?* Never smokers are those who answered no to the first question, former smokers are those who answered yes to the first question then “not at all,” and current smokers are those who smoke both some days and every day. Current alcohol use is defined as participants who reported having at least one drink of any alcoholic beverage in the past 30 days.

The county of residence for each respondent was used to create a new variable indicating region of residence. The three regions we examined were the Appalachian region, as defined by the Appalachian Regional Commission (Appalachian Regional Commission 2022), the Delta region, as defined by the Delta Regional Authority (Delta Regional Authority 2023), and Central Kentucky. For participants who were missing county information, we used an imputed region variable from the BRFSS to ascertain the region of residence. The BRFSS imputed region variable represents the following six regions of Kentucky: Bluegrass, Central, Eastern, KIPDA, Northern, and Western. While there is some overlap between our three regions and the six imputed BRFSS regions, the KIPDA and Northern regions contain only counties located in what we defined as Central Kentucky. These two imputed regions were used to define the region of residence for some participants in Central Kentucky. At the time this data was collected, only one state bordering Kentucky had legal recreational use and sales (Illinois). Three bordering states had legal medical use (Ohio, Virginia, and West Virginia). Virginia legalized recreational use in July 2020, though recreational sales have yet to begin.

### Data analysis

We conducted all analyses using SAS 9.4 (SAS Institute, Cary, NC), and followed CDC guidelines to ensure proper handling of the complex sampling design and survey weights to produce population-based prevalence estimates across the two years of pooled data (CDC 2022a). The use of the survey weights is necessary to make generalizations from the sample to the population, as they adjust for noncoverage and nonresponse and force the total number of cases to equal population estimates for each geographic region (CDC 2022b). We performed sensitivity analysis of different classifications of heavy use compared to daily use, using a range of at least 15–29 days. Cross-tabulation was used to assess cannabis use prevalence overall by demographic, socioeconomic, and geographic factors, and to assess modes of use and reason for use by the same. We also calculated age-adjusted rates of cannabis use for the Appalachian and Delta regions, using Central Kentucky as the reference population. Chi-squared tests were used to assess differences in these demographic, socioeconomic, and geographic factors by cannabis use status.

As a comparison to Kentucky’s overall prevalence of cannabis use, we calculated the total prevalence of cannabis use in other states where it was also illegal for any purpose. For 2020, this included Idaho, Indiana, Mississippi, South Carolina, Tennessee, and Wyoming, and for 2021, Idaho, Indiana, and Wyoming. We also calculated their modes and reasons for using cannabis. We chose these states because they are the only ones, besides Kentucky, where cannabis was illegal for any purpose at the time of data collection and used the BRFSS optional cannabis use module.

Lastly, logistic regression was used to examine predictors of cannabis use before and after adjustment for all other factors examined here. We assessed multicollinearity among the predictor variables using several methods. The Pearson correlation coefficients were computed to identify highly correlated pairs of variables. Variance Inflation Factors (VIFs) were calculated for each predictor, with VIF values exceeding 5 considered indicative of multicollinearity. Additionally, we examined the condition index, with values above 30 suggesting potential multicollinearity.

## Results

**Fig. 1:**
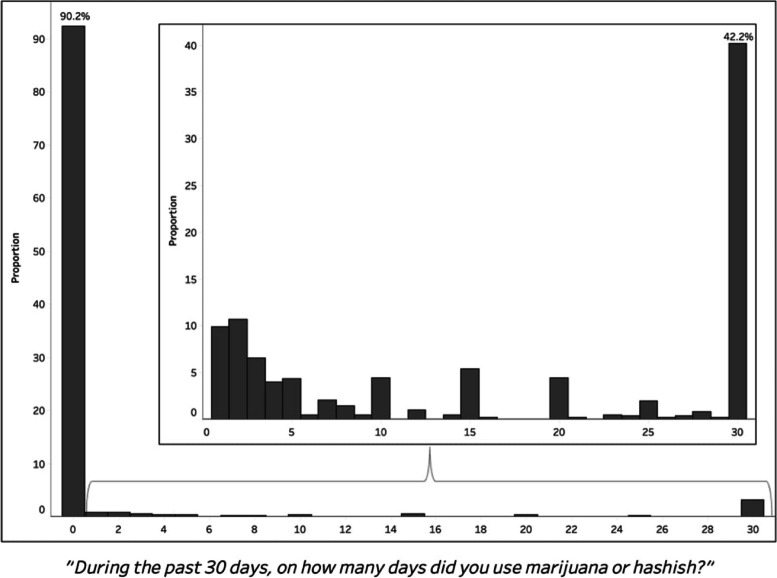
Distribution of number of days of reported cannabis use in KY, 2020–21

In 2020 and 2021, there were a total of 9363 BRFSS respondents in Kentucky. Of these, 8175 provided information for past 30-day cannabis use and thus comprised the sample for this analysis. Results of analysis implementing the sampling weights showed that the majority of participants (90.2%) reported zero days of cannabis use in the past 30 days (Fig. 1). Of those who report using cannabis in the past 30 days, 42.2% reported using it for all 30 days. We defined heavy use as at least 20 of the past 30 days, which comprised 49.2% of people who use cannabis, and 4.8% of the total sample. Cannabis use rates were very similar in other states where it is illegal for any purpose (9.2%), though slightly lower than Kentucky.

The observed p-values and associations between different factors and cannabis use status were very similar when heavy use was classified as between at least 20 to 30 days of use. Only one participant reported between 16 to 19 days of use. Estimates change slightly when heavy use was classified as at least 15 days of use. These results indicate that the classification of at least 20 days of use in the past 30 days is a robust measure for heavy cannabis use.

### Demographics of cannabis use

Overall, the past-month prevalence of cannabis use among Kentucky adults was 9.8% (Table 1). Cannabis use rates were very similar, though slightly lower, in other states where it is illegal for any purpose (9.2%). The prevalence of use was higher in males (12.3%) than females (7.5%). Those aged 18 to 34 were most likely to use cannabis (16.1%), with prevalence decreasing progressively among older age groups. Participants in the lowest educational attainment category (high school graduate or less) reported the highest rate of use (10.9%); this is the only education category that reported higher rates of heavy (6.2%) than occasional (4.7%) use. Those without healthcare coverage reported significantly more use (18.1%) than those with coverage (9.2%) and had a higher proportion of heavy (11.7%) than occasional (6.4%) use.

**Table 1:**
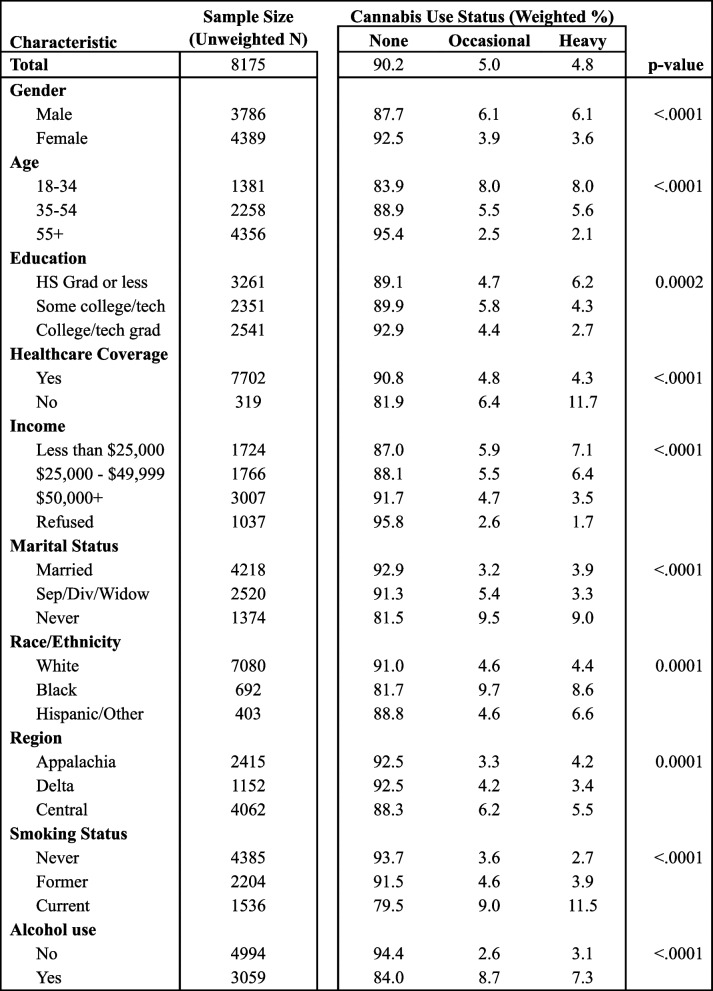
Cannabis use in KY by demographic, socioeconomic, and geographic factors, 2020-21

Chi-squared *p*-values are reported

Use varied somewhat among income groups, with rates of cannabis use decreasing as income increased. Rates were similar between the lower two income groups, with these two groups also reporting higher rates of heavy use than occasional use. Those who were currently married or cohabitating had similar rates of use to the those who were separated, divorced, or widowed (7.1% and 8.7%, respectively), though the married or cohabitating participants had a higher proportion of heavy than occasional users. Those who were never married reported the highest rates of use (18.5%). Cannabis use was least common in white people (9.0%) and highest for black people (18.3%).

Participants who currently smoke cigarettes have much higher rates of cannabis use (20.5%) than their former (8.5%) and never (6.3%) smoking counterparts. Among the three categories of smoking status, current smokers are the only ones who reported a higher rate of heavy (11.5%) than occasional (9.0%) cannabis use. People who consumed at least one alcoholic beverage in the past 30 days had a higher proportion of overall cannabis use (16.0% compared to 5.6%), however, those who did not consume any alcohol had a higher proportion of heavy use than occasional use.

**Fig. 2:**
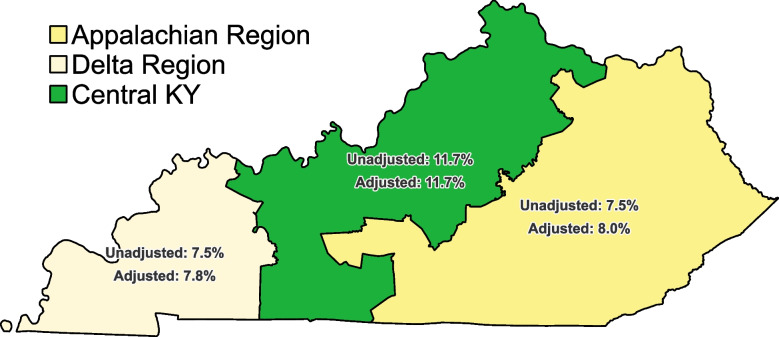
Age-adjusted prevalence of cannabis use by region in KY, 2020–21

Residents of Kentucky’s Appalachian and Delta regions had the same rate of cannabis use (7.5% each), with the highest prevalence being in Central Kentucky (11.7%). After adjusting for age differences between regions, the prevalence was still highest in Central Kentucky (11.7%), followed by the Appalachian (8.0%) then Delta (7.8%) regions (Fig. 2). Table 2 shows the age and race/ethnicity distribution among those who used cannabis. The distribution by age group was very similar for the Appalachian and Central regions, but those who used cannabis in the Delta region were more likely to be 55 + , though this was not statistically significant.

**Table 2:**
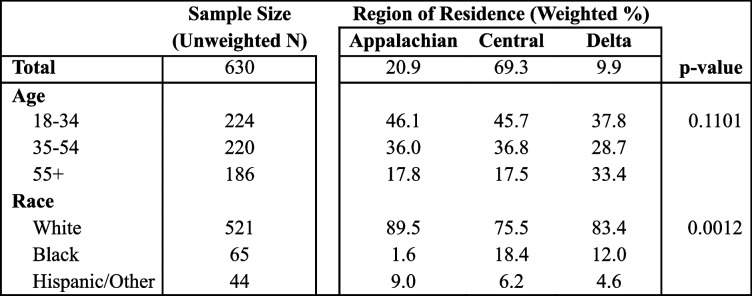
Age and race/ethnicity of Kentuckians who use cannabis by region, 2020-21

Chi-squared *p*-values are reported

### Mode preferences

Modes of use varied by age group, education level, income group, marital status, and reason for use (Table 3). Smoking was by far the most common mode of use for all demographic and socioeconomic groups (63.5%−87.0%, 77.5% overall). Eating or drinking was the second most reported mode (9.1%−22.9%, 14.0% overall) and concentrates were the third most common mode (4.1%−15.2%, 7.3% overall) for all respondents. Married and cohabitating adults reported the highest rates of eating or drinking their cannabis (18.2%) compared to those who were never married (10.7%). Those who reported earning $50,000 + were more likely to eat or drink cannabis (22.9%) than the lower income groups.

**Table 3:**
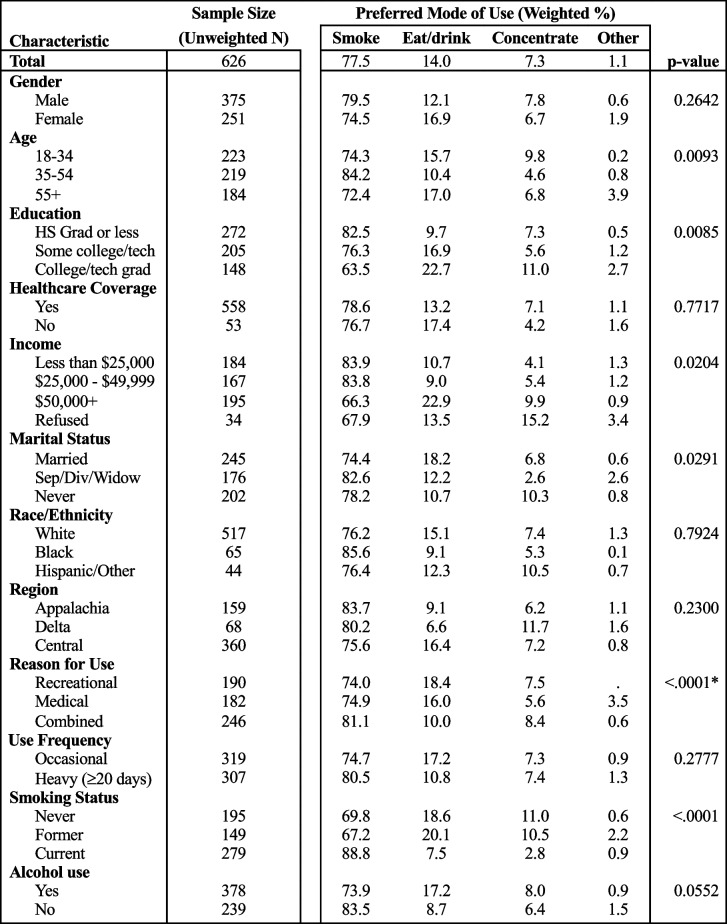
Cannabis use preferred mode in KY by demographic/socioeconomic factors, 2020-21

The total *N*=626 due to four participants who used cannabis missing data for preferred mode, and each covariate total may not equal 626 due to missing data for that covariate. Chi-squared *p*-values are reported

*Computed using Agresti’s method for cell with 0 count (Agresti 1992)

Concentrate use was highest for the 18 to 34 age group (9.8%), college or technical school graduates (11.0%), higher income groups, and those who were never married (10.3%). Other modes of use were preferred by very few people who use cannabis, with the exception of those aged 55 + (3.9%) and those who used cannabis for medical reasons only (3.9%). Mode of use did not vary significantly by race/ethnicity, region of residence, or frequency of use. Other states where cannabis is illegal had slightly lower rates of smoking (77.0%) and ingesting (10.6%) cannabis, and slightly higher rates of concentrate use (8.9%) and other modes not listed (3.4%) than Kentucky.

### Reasons for cannabis use

Overall, 24.4% of respondents who used cannabis reported using it for medical reasons only, 32.8% for recreational reasons only, and 42.8% for both reasons (Table 4). Reasons for use varied by gender, age group, education level, healthcare coverage status, marital status, frequency of use, and alcohol use status. Females were more likely to use cannabis for medical reasons alone (30.5%) and males were more likely to use for recreational reasons alone (36.0%). Participants aged 55 + reported the highest rate of using cannabis for medical reasons alone (46.2%) compared to other age groups. In other states where cannabis was illegal for any reason, there was a higher proportion of people using cannabis for recreational reasons alone (37.8%) and a lower proportion of people using for both medical and recreational reasons (37.3%) than in Kentucky.

**Table 4:**
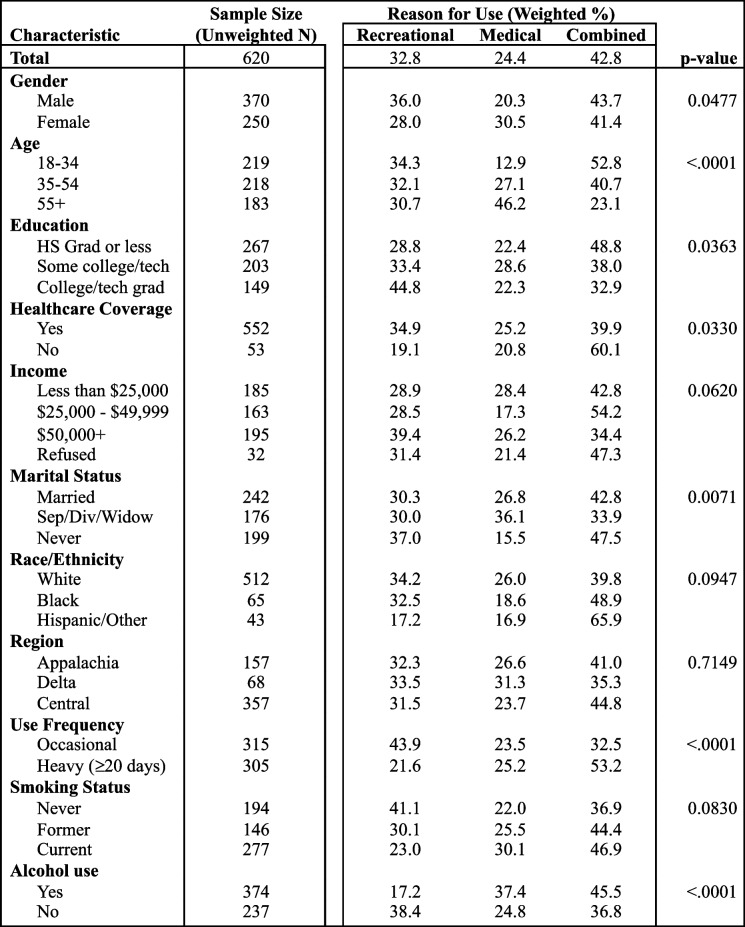
Reasons for cannabis use in KY by demographic/socioeconomic factors, 2020-21

The total *N*=620 due to 10 participants who used cannabis missing data for reason for use, and each covariate total may not equal 620 due to missing data for that covariate. Chi-squared *p*-values are reported

Those aged 18–34 reported the highest rate of both medical and recreational reasons for use (52.8%). College or technical school graduates reported higher rates of recreational-only use (44.8%) than other education levels. The majority of those without healthcare coverage who used cannabis reported doing so for both medical and recreational reasons (60.1%). Participants who reported having healthcare coverage had higher rates of recreational use than those without healthcare coverage (34.9% and 19.1%, respectively).

Respondents who have never been married reported the highest rate of cannabis use for recreational reasons only (37.0%) and the lowest rate of medical reasons only (15.5%) compared to those of other marital statuses. Those who were separated/divorced/widowed reported the highest rate of medical reasons only (36.1%). The majority of participants who reported using cannabis daily or near daily reported doing so for both medical and recreational reasons (53.1%). People who reported using cannabis for 1–19 of the past 30 days had a higher rate of using it for recreational reasons only (41.8%) compared to those who reported using cannabis daily or almost daily (20.9%). Those who consumed at least one alcoholic beverage in the past 30 days reported lower rates of using cannabis for recreational reasons only (17.2%) than those who did not consume any alcohol (38.4%).

### Regression analysis

For this logistic regression analysis, the outcome of interest was any cannabis use (at least once in the past 30 days) compared to none (Table 5). After adjustment for all socioeconomic and demographic factors, people who use cannabis had significantly higher odds of being male (aOR = 1.53, 95% CI 1.19 – 1.97) as well as having never been married (aOR = 1.58, 1.13 – 2.21). We observed an inverse relationship between age and cannabis use, which remained significant for all age groups both before and after adjustment. After adjustment, Black Kentuckians remained more likely to use cannabis (aOR = 1.94, 1.26 – 2.97) than white Kentuckians, though this association was attenuated. People who used cannabis in the past 30 days were nearly three times more likely to also have consumed alcohol in the past 30 days (aOR = 2.90, 2.22 – 3.80). The association between cigarette smoking status and cannabis use became stronger after adjustment, with both current (aOR = 4.83, 3.52 – 6.61) and former smokers (aOR = 2.22, 1.59 – 3.10) being more likely to use cannabis than never smokers. Those who use cannabis had lower odds of living in either the Appalachian (aOR = 0.60, 0.45 – 0.80) or Delta (aOR = 0.57, 0.40 – 0.83) regions of Kentucky than the Central region. The differences between estimates in the unadjusted vs adjusted model are most prominent in healthcare coverage, marital status, race, and cigarette smoking status.

**Table 5:**
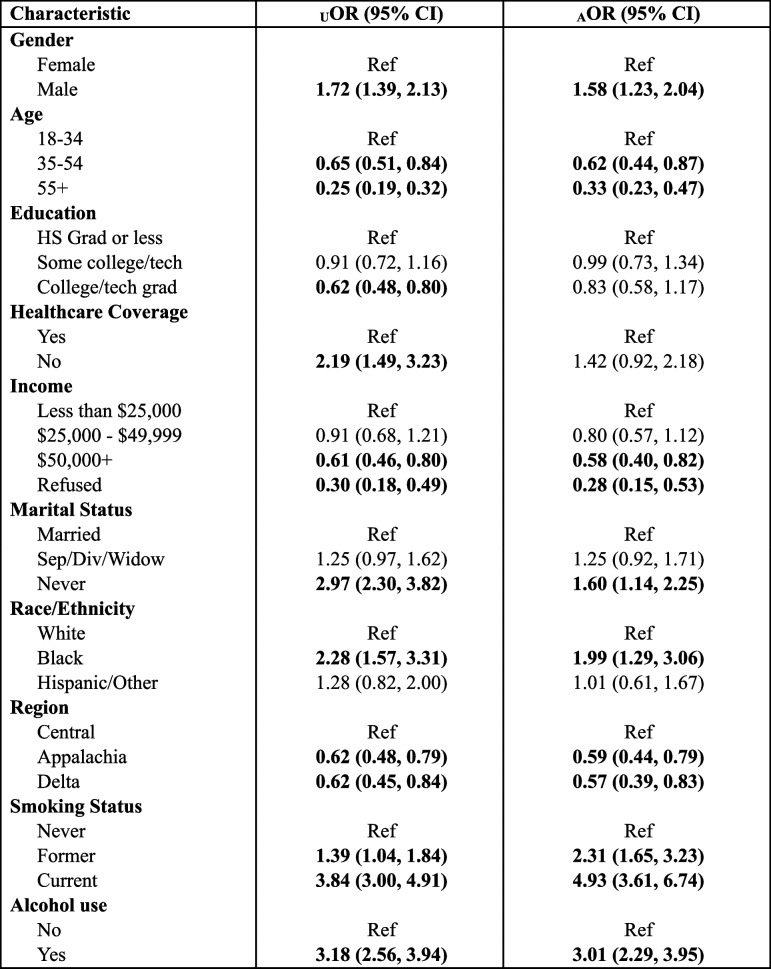
Logistic Regression predicting past 30-day cannabis use in KY, 2020-21

*U* Unadjusted, *A* Adjusted for all other factors, *OR* Odds ratio, *CI* Confidence interval

There was no evidence of multicollinearity, however there was evidence of an interaction between healthcare coverage and income level. There were zero participants who reported using cannabis, refused to share their income, and did not have healthcare coverage. When those who refused to disclose their income level were removed from analysis, this interaction dissipated.

## Discussion

The present study provides an important update to the scope of cannabis use in Kentucky. Overall, past-month cannabis use rates were lower in Kentucky (9.8%) than national estimates for 2021 (13.0%) (Substance Abuse and Mental Health Services Administration (SAMHSA), 2022). Despite its illicit status, prevalence of use is similar among population subgroups, with some variation in each. Nearly 12% of those in Central Kentucky reported using cannabis at least once in the past month, compared to only 7.5% in the Appalachian and Delta regions. This variation is partly due to differences in age and race distributions of the populations, which might be explained by three large and relatively diverse cities in Kentucky (Louisville, Lexington, and Bowling Green) being in Central Kentucky (Census 2023). We expected to see higher rates of cannabis use in the Delta region due to its proximity to the legal market in Illinois (the only one in the region in 2020–2021). This was not the case, however, except that a larger share of cannabis users in the Delta region were 55 or older (Table 2), which could suggest higher rates for medical use, although this was not a significant difference. There was also notably lower variation in preferred mode of consumption for these two regions compared to the Central region. These prevalence of use differences remained after adjustment for differences in age distributions.

There are some possible explanations for why cannabis use rates in Kentucky are lower than national estimates, further than the simple fact that it was illegal for any purpose with no pending legislation when this data was collected. Some hemp-derived cannabinoids, such as Δ−8 THC, that are federally legal can produce intoxicating effects similar to Δ−9 THC (cannabis). As previously mentioned, Δ−8 THC and other hemp-derived cannabinoids became de facto legal under the 2018 Farm Bill. The popularity of Δ−8 THC grew dramatically in late 2020, and as of early 2021, became considered one of the fastest-growing segments of hemp derived products (Kruger & Kruger, 2022). In a sample collected in 2021, adults in the US who used Δ−8 THC had higher odds of living in states with no cannabis legalization compared to those living in states with either medical-only or full (medical and recreational) cannabis legalization. It is possible that novel cannabinoids are being used in place of cannabis, since they are more accessible and the two produce similar effects (Kruger & Kruger, 2022). Use of novel cannabinoids was not examined here, but further research should be conducted to examine their prevalence and modes of use.

Matching previous studies, smoking was the most common mode of use, with nearly 78% of all respondents reporting it as their preferred mode (Schauer et al. 2020). Despite the lack of legal retail options, other modes of consumption were relatively common overall as well, especially among those with higher education and income. There was notably low variation in preferred mode among Black Kentuckians and those in the middle (35–54 years) age group. The youngest age group (18–34 years) had the highest rate of concentrate use, while those in the oldest age group (55 +) reported the highest rate of a mode not listed. Modes of consumption not listed might include products such as tinctures and topicals. It may be important to distinguish those who use topicals specifically as they do not produce psychoactive effects like other THC products.

There were notable differences of preferred mode by marital status, with married respondents reporting the highest rates of eating or drinking their cannabis. Consuming cannabis in food or drinks is more discreet than other modes since it does not involve burning of the product and does not produce smoke like most others (Barrus et al. 2016); it may be worthwhile to investigate whether the presence of children mediates the association between marital status and preferred mode of use. This may be important because most cases of marijuana intoxication involve young children (of toddler age) and the unintentional ingestion of high-potency edible products (Diebold 2017). Further consideration should also be given to people who use multiple modes of consumption, which the BRFSS does not currently collect data on.

Continued surveillance of cannabis use in Kentucky is warranted, particularly to see how new legislation affects the scope of use. Smoking has always been the most popular mode, with the highest rates of non-smoking modes being in states where cannabis is legal compared to states where it is not (Goodman et al. 2020). In coming years, it is expected that Kentucky will see a higher prevalence of cannabis use and more variation in preferred mode of use, particularly after legal sales begin in the state and different products become more accessible.

Overall, more than half of people (67.2%) who use cannabis report doing so for a medical reason, with 24.4% using for medical reasons only. Women and older individuals reported higher rates of using cannabis for medical reasons. Those with higher levels of education and those who have never been married reported higher rates of using cannabis for recreational reasons only. There was no variation in reason for use between income, racial, and regional groups. The proportion of those who use cannabis for recreational reasons only is slightly higher in other states where cannabis is illegal (37.8%). Further research should be conducted to determine which medical reasons or conditions the general population reports using cannabis for.

Updated surveillance on cannabis use is important given the ever-changing legal landscape as well as differences in potency of cannabis products today compared to just ten years ago. There has been a call for updated measures of cannabis use that include both frequency and potency when analyzing its associated risks and benefits. This study provides a basis of understanding for how these measures relate to each other and other factors.

## Limitations

This study contains limitations that must be taken into consideration with its results. First, the BRFSS is a self-reported survey asking questions related to illicit drug use. Respondents may not be entirely honest in their answers to questions about cannabis. We lack data about multimodal use because this survey only asks about the most frequent mode of use. This survey does not collect data from the incarcerated population, which almost certainly has higher rates of lifetime cannabis use. This slightly limits who the results are generalizable to.

Second, 2020 had the lowest response rates for the BRFSS nationwide due to the COVID-19 pandemic (CDC 2021). The pandemic led to a major shift in the work force, starting in March of 2020. Some of the BRFSS data collectors were forced to cease work due to logistical difficulties and could not make calls for some time (CDC 2021). Kentucky was not able to conduct surveys for each of the 12 months of 2020 and did not begin data collection until May (CDC 2021). While it did meet the minimum requirements to be included in the 2020 BRFSS public-use data set, there might be differences in estimates and analysis when compared to other years (CDC 2021).

Third, the national estimate for past month cannabis use cited (13%) includes adolescents aged 12–17 (SAMHSA, 2022), which is not a direct comparison to our study that includes adults only. However, the percentage of people who used cannabis in the past month was highest among young adults aged 18 to 25 (24.1%), followed by adults aged 26 or older (12.2%), then by adolescents aged 12 to 17 (5.8%) (SAMHSA, 2022). We believe the prevalence for past month cannabis use for adults (aged 18 + years old) in the US is higher than the cited estimate.

Finally, 2020 and 2021 are the only years that the state of Kentucky has used the optional marijuana use module. This prevents any sort of analysis to establish any sort of trends or comparisons to previous years. Because the marijuana use module was not used in 2022 or 2023, estimates presented here may be biased if used as a pre-legalization baseline. We suspect that the prevalence of cannabis use has in Kentucky has slightly increased over the last couple years and will continue to do so post-legislation.

## Conclusion

Overall, cannabis use rates in Kentucky remain lower than national estimates, but similar to other states where cannabis is illegal for any purpose. Despite the illegal status of cannabis in Kentucky, the five most common modes of use (smoking, eating, drinking, vaping, dabbing) were present among population subgroups. As nearby states legalize cannabis, the pandemic restrictions disappear, and Kentucky’s own new medical cannabis markets open, we speculate that use of cannabis and experimentation with non-smoking modes of use may increase. Generally, the low number of respondents in the BRFSS and the COVID-19 pandemic may have limited this study’s findings, but it remains the most up-to-date examination of cannabis use among adults in Kentucky.

## Data Availability

Most data are publicly available through the CDC at https://www.cdc.gov/brfss/annual_data/annual_data.htm. A request for the county variable was granted from the Kentucky Department for Public Health.

## Abbreviations

AIDS: Acquired immunodeficiency syndrome
BRFSS: Behavioral Risk Factor Surveillance System
CBD: Cannabidiol
CDC: Centers for Disease Control and Prevention
CI: Confidence interval
COVID-19: Coronavirus disease
KY: Kentucky
OR: Odds ratio
THC: Tetrahydrocannabinol
US: United States
VIF: Variance inflation factor
